# Subtractive panning for the isolation of monoclonal PEPITEM peptide antibody by phage display

**DOI:** 10.1038/s41598-023-40630-7

**Published:** 2023-08-21

**Authors:** Mohammed Alassiri, Jing Yi Lai, Angela Chiew Wen Ch’ng, Yee Siew Choong, Asma Alanazi, Theam Soon Lim

**Affiliations:** 1 Department of Basic Sciences, College of Science and Health Professions, King Saud bin Abdulaziz University for Health Sciences (KSAU-HS), Riyadh, Kingdom of Saudi Arabia; 2 King Abdullah International Medical Research Center (KAIMRC), Riyadh, Kingdom of Saudi Arabia; 3 Department of Pathology and Laboratory Medicine, King Abdulaziz Medical City (KAMC), Ministry of the National Guard - Health Affairs, Riyadh, Kingdom of Saudi Arabia; 4 Institute for Research in Molecular Medicine, Universiti Sains Malaysia, 11800 Minden Penang, Malaysia; 5 College of Medicine, King Saud bin Abdulaziz University for Health Sciences (KSAU-HS), Riyadh, Kingdom of Saudi Arabia; 6 Analytical Biochemistry Research Centre, Universiti Sains Malaysia, 11800 Minden Penang, Malaysia

**Keywords:** Biotechnology, Molecular biology

## Abstract

Antibody phage display is a key tool for the development of monoclonal antibodies against various targets. However, the development of anti-peptide antibodies is a challenging process due to the small size of peptides for binding. This makes anchoring of peptides a preferred approach for panning experiments. A common approach is by using streptavidin as the anchor protein to present biotinylated peptides for panning. Here, we propose the use of recombinant expression of the target peptide and an immunogenic protein as a fusion for panning. The peptide inhibitor of trans-endothelial migration (PEPITEM) peptide sequence was fused to the *Mycobacterium tuberculosis* (Mtb) α-crystalline (AC) as an anchor protein. The panning process was carried out by subtractive selection of the antibody library against the AC protein first, followed by binding to the library to PEPITEM fused AC (PEPI-AC). A unique monoclonal scFv antibodies with good specificity were identified. In conclusion, the use of an alternative anchor protein to present the peptide sequence coupled with subtractive panning allows for the identification of unique monoclonal antibodies against a peptide target.

## Introduction

Since the advent of hybridoma technology, the development of recombinant antibody technology has gone through several key technological inceptions. Phage display is regarded as one of the key approaches used for the discovery of novel monoclonal antibodies against various targets of interest. The introduction of display technologies like phage display has helped to reduce the dependency on animal hosts for antibody development making the process somewhat “greener”. A detailed review on antibody phage display technology has been provided by Valldorf, et al.^1^ and Lai and Lim^2^.

The application of antibody phage display for the development of antibodies against protein targets is well documented. However, the application of phage display to isolate antipeptide antibodies remain scarce in comparison to protein antigens. The complexity of isolating antibodies against peptides is mainly due to the conformational flexibility of peptides, together with the subsequent loss of entropy when bound by antibodies^3^. Even so, there have been some reports highlighting the use of antibody phage display to generate antibodies against peptide targets^4^. Development of anti-peptide antibodies have been carried out using natural sourced naïve^4^ as well as engineered synthetic antibody libraries^5^.

The main principle for the isolation of target specific antibodies is through a process consisting of several key steps commonly known as biopanning. The steps include target immobilization, antibody binding, washing and rescue. This repetitive cycle of elimination allows for the concentration of a specific species of antibody clones after 3 to 5 rounds. The selection and segregation process in biopanning is guided by the affinity of the antibody against the target present. A recent review highlighted the different approaches for target presentation during panning including the different strategies and considerations associated to the success of a panning campaign^6^. One panning strategy that is of interest is the use of subtractive panning approach where competition with the target antigen is carried out by a control target with high similarity to enrich antibodies that target a specific location of a target. A modified version of such a panning process called Ying-Yang panning was done showcasing the possibility to isolate target specific monoclonal antibodies using crude protein preparation by deselecting with a control crude protein preparation^7^.

Due to their short and small nature, peptide targets are difficult to produce independently in vivo. Therefore, synthetic peptides are the common choice as targets for panning campaigns. However, the synthetic peptides would still need to be chemically conjugated to a larger anchor protein to ensure optimal presentation for antibody binding. Synthetic peptides are normally conjugated either with bovine serum albumin (BSA), ovalbumin (OVA) or keyhole limpet hemocyanin (KLH) for antibody development^8,9^. Additionally, there is the option of biotinylation where biotinylated peptides are bound to streptavidin for panning. These approaches may not be suitable sometimes as chemical conjugation can result in modifications to the peptide structure. The cost to synthesize peptides with modifications for conjugation or biotinylation could also pose a challenge for certain laboratories.

**Figure 1:**
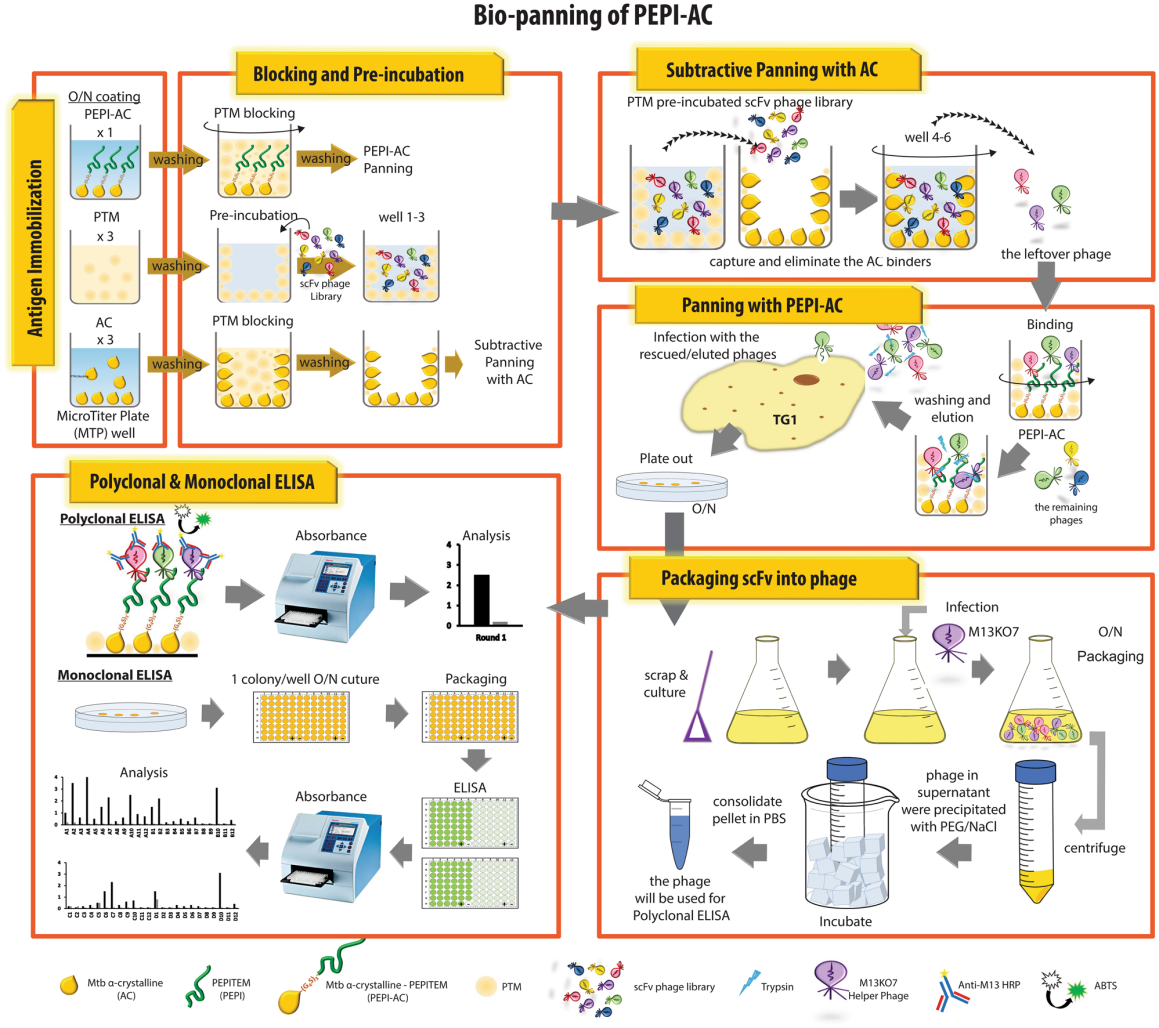
The outline of the subtractive panning campaign. PEPI-AC, AC and PTM were immobilized overnight on microtiter plate well. The library phage was pre-incubated with PTM in PTM coated well and the process was repeated twice (well 1–3). Subsequently, the pre-incubated library phage was transferred to AC coated well to capture and eliminate AC binders from the library phage pool. The process was repeated twice (well 4–6). Lastly, the library phage was transferred to PEPI-AC coated well to isolate specific binders. Bound phage was eluted and recovered by infecting TG1 and plate out on ampicillin supplemented agar plate. The colonies were scraped and culture in 10 mL 2 × YT supplemented with ampicillin and glucose. The culture was infected with M13KO7 helper phage when OD_600nm_ 0.5 was reached. After overnight packaging, the culture was pellet down and phages secreted in the medium were precipitated with PEG/NaCl. The recovered phages were resuspended with PBS and use for polyclonal and monoclonal ELISA.

In this study, the target utilized was PEPITEM (PEPtide Inhibitor of Trans-Endothelial Migration). PEPITEM is a B-cell secreted linear peptide derived from 14 to 3–3 protein zeta/delta and is functioned to inhibit T-cells trafficking into inflamed tissue. This inhibitory effect is achieved through promoting synthesis of sphingosine-1 phosphate when the PEPITEM peptide binds to cadherin-15 on endothelial cells^10,11^. Despite multiple attempts by our laboratory to enrich antibodies against the PEPITEM peptide using a biotinylated synthetic peptide construct, the panning was unsuccessful with no enrichment of binders or enrichment of background binders (Supplementary Fig. S1). Challenges to enrich anti-peptide antibodies could be due to the antigenicity and presentation issues as mentioned earlier. As such, we attempted an alternative peptide presentation method for antibody panning campaigns. Here, we synthesized the DNA sequence for PEPITEM peptide and fused it to the *Mycobacterium tuberculosis* (Mtb) α-crystalline (AC) as an anchor protein. The peptide is connected to the anchor protein via a flexible linker to allow flexibility for the peptide to maintain a more natural conformation in the fusion state (PEPI-AC). Panning was carried out with the fusion construct and subtractive selection was done using a non-fused AC protein (Fig. 1). The process was able to yield successful isolation of monoclonal antibodies specific against PEPITEM in both phage and soluble form. Therefore, the adaptation of a peptide-anchor protein approach can be utilized to identify antipeptide monoclonal antibodies.

## Results

### Cloning and expression of AC and PEPI-AC

PEPITEM (SVTEQGAELSNEER) is a linear epitope of the human protein, 14–3–3 protein zeta/delta (UniprotKB: P63104). The DNA sequence corresponding to residue S28-R41 was obtained from GenBank (NM_001135699.2) and synthesized as a forward and reverse primers. Both primers were assembled and cloned to 5’ end of AC gene. A short glycine-serine (GS) rich linker was included in between the PEPITEM and AC gene to allow some flexibility. In total, two gene constructs were cloned into pRSET-pelB vector: PEPI-AC served as the target antigen while AC served as subtractive antigen to eliminate non-specific binders to it.

**Figure 2:**
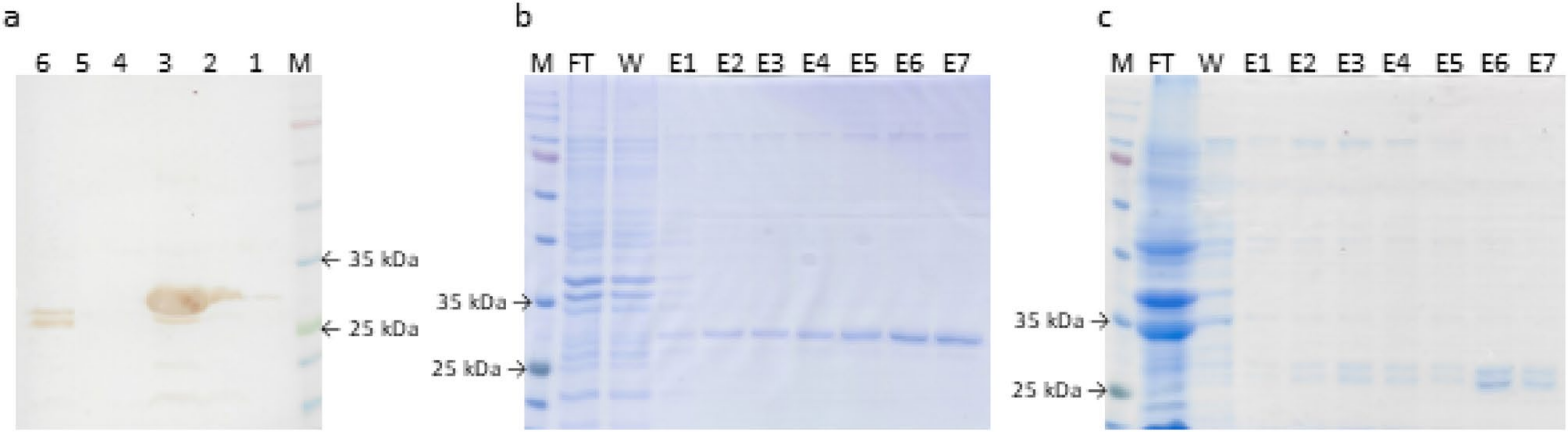
Expression of PEPI-AC and AC. (**a**) Western blot of the protein extracted from different fraction. M: Opti-XL protein marker (abm), 1: PEPI-AC periplasmic crude, 2: PEPI-AC cytoplasmic crude, 3: PEPI-AC pellet, 4: AC periplasmic crude, 5: AC cytoplasmic crude, 6: AC pellet. The proteins were probed with Streptavidin HRP (1:5000). Majority of the proteins were remained in the pellet. Purification of (**b**) PEPI-AC and (**c**) AC were analysed on 12% SDS-PAGE. M: Opti-protein XL marker (abm), FT: flow-through, W: wash, E1-7: elution fraction 1–7. The original blot and gel images are presented in Supplementary FigS. S2–S4.

PEPI-AC and AC were expressed using BL21 (DE3). The culture was induced with 1 mM IPTG at OD_600nm_ of 0.6 and supplemented with 50 μM biotin to produce biotinylated AC and PEPI-AC. After overnight expression at 25 °C, extraction of AC and PEPI-AC in periplasmic and cytoplasmic fraction were attempted, but the yield of both proteins were relatively low, and the yield from purification was low. As demonstrated in the western blot (Fig. 2a), majority of the protein remained in the pellet. Hence, AC and PEPI-AC were extracted using urea buffer and subsequently eluted using imidazole buffer. Figure 2b and c shows the purified fraction of PEPI-AC and AC analysed on 12% SDS-PAGE. There are two bands observed in the purified fraction of AC. Both the bands were detected in western blot as shown in Fig. 2a. The two bands could represent partial cleavage of pelB which should theoretically be cleaved off during transportation of the protein across the membrane to the periplasm^12^ or degradation. Since the protein was accumulated in the cytoplasm as inclusion protein, it could contain both pelB cleaved and un-cleaved proteins. Nonetheless, this does not defeat the purpose of AC protein as a subtractive antigen to remove non-specific binders to the AC protein in PEPI-AC panning campaigns. The expected size of the PEPI-AC and AC are of the same size at approximately 25 kDa. The AC was designed with a peptide insertion to maintain size similarity to PEPI-AC to be used as a subtractive substrate during panning. Although the expected size was approximately 25 kDa, the bands were visibly migrating at a higher band size of approximately 27 kDa which could be due to the biotinylation of the proteins^13,14^.

### Isolation of PEPITEM specific monoclonal antibodies

**Figure 3:**
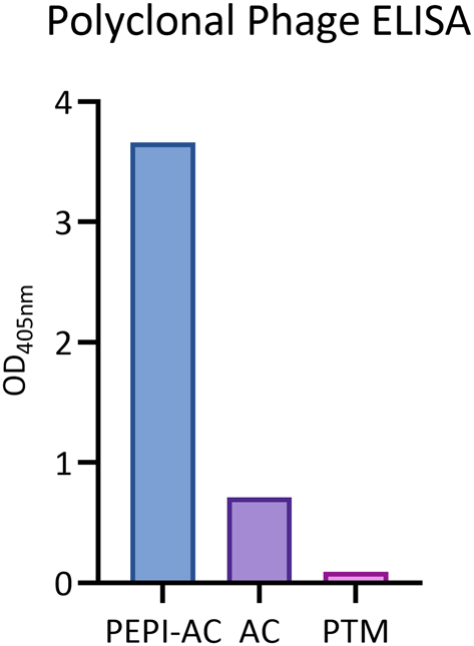
Polyclonal phage ELISA for PEPI-AC subtractive panning. AC and PTM were served as background controls and showed lower signal compared to PEPI-AC. The absorbance reading of ABTS at OD_405nm_ were plotted.

To isolate antibodies specific to PEPITEM, subtractive panning was carried out where 10^11^ cfu of naïve library phage was pre-incubated in wells coated with milk and AC, respectively, prior to binding with PEPI-AC. A total of 2 × 10^3^ cfu phage was rescued and subsequently re-amplified using TG1. Polyclonal ELISA showed absorbance reading (OD_405nm_) of 3.663, 0.713 and 0.093 for PEPI-AC, AC and milk respectively (Fig. 3). AC and milk serve as background for non-specific interaction analysis. The signal from a single round of panning yielded PEPI-AC signals of 5- and 39-fold higher than AC and milk respectively. This indicated sufficient enrichment for monoclonal antibody identification.

**Figure 4:**
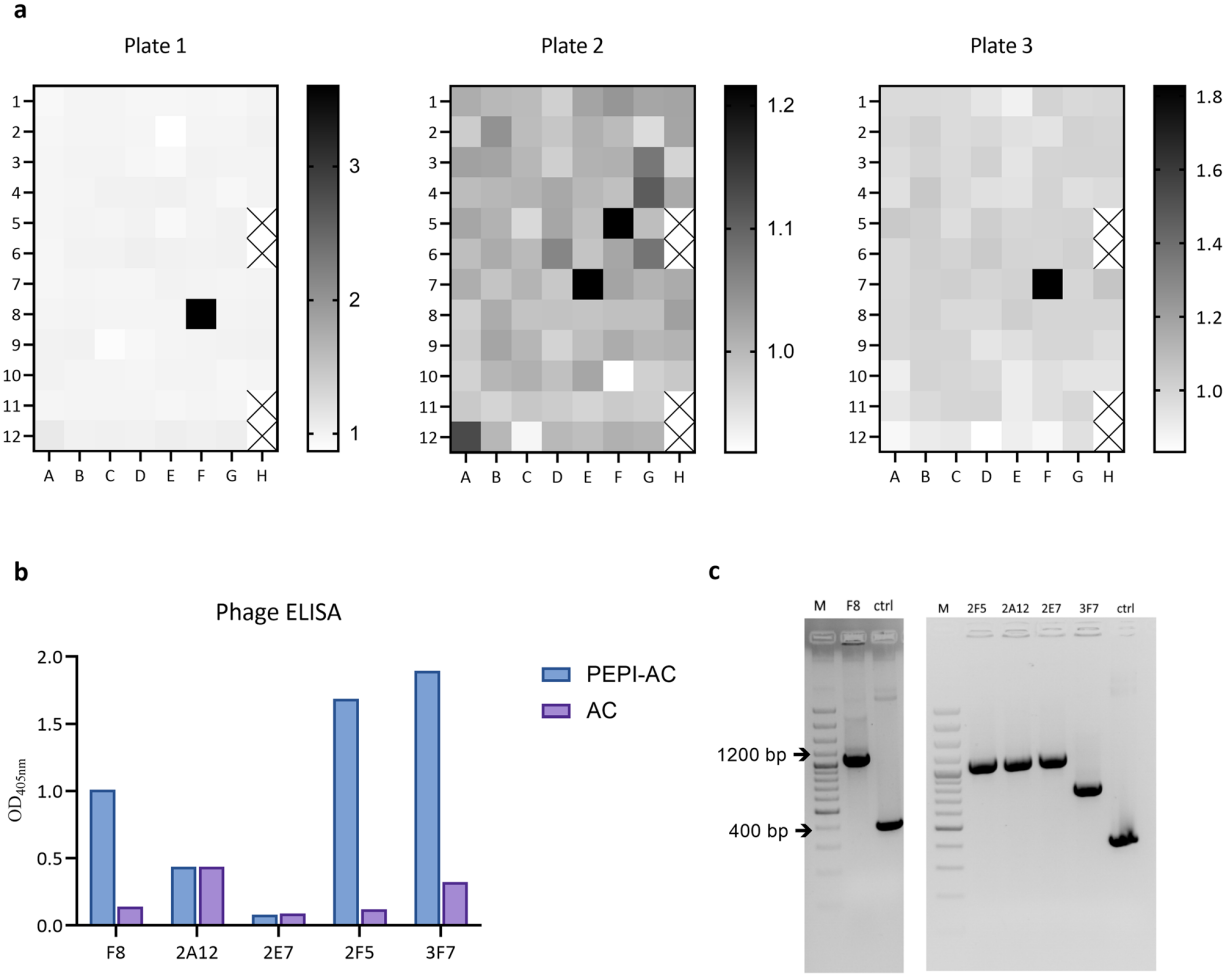
Selection of PEPI-AC specific monoclonal scFv. (**a**) Screening of monoclonal antibodies against PEPI-AC in 96-well plate. The signal (PEPI-AC) over noise (AC) reading were demonstrated in the heatmap, with in-house ELISA control (H5, H6, H11, H12) excluded from the analysis. The clone with relatively higher signal in the three independent screening were picked and re-packaged to test in phage ELISA. (**b**) Phage ELISA of clone F8, 2A12, 2E7, 2F5 and 3F7 were performed. The signal against background (AC) was higher for clone F8, 2F5, and 3F7, whereas clone 2A12 and 2E7 did not show difference in the signal between PEPI-AC and AC. (**c**) The clones were subjected to colony PCR and showed that all clones containing full scFv size (1200 bp), except for clone 3F7 which is only half of the size. M represents 100 bp plus DNA ladder (Thermo Scientific) and ctrl represents the pLABEL vector. The original gel image is presented in Supplementary Fig. S5.

After the subtractive panning procedure, monoclonal antibodies were screened from the polyclonal phage population. Three 96-well plates were screened with a total of 140 clones tested. Figure 4a shows the monoclonal phage ELISA results from the clones. The signals are presented as ratio of PEPI-AC signal over AC signal. Clone F8 from plate 1, clone 2A12, 2E7 and 2F5 from plate 2, clone 3F7 from plate 3 showed relatively higher signals on their respective plates. These clones were repackaged and tested in phage ELISA (Fig. 4b). Clone F8, 2F5 and 3F7 showed a good signal on PEPI-AC coated well and low signal on AC and BSA coated wells. The 5 clones were also checked on colony PCR (Fig. 4c). Although, clones F8, 2F5 and 3F7 showed good binding but only clone F8 and 2F5 showed complete scFv size (approximate 1200 base pair (bp) in colony PCR). Hence, F8 and 2F5 were sent for Sanger sequencing to retrieve the antibody gene sequence.

### Characterization of the monoclonal antibodies

The DNA sequence of the clone F8 and 2F5 were analysed on IMGT/V-quest. Both clones belong to IGHV3 and IGLV2 gene families, with different V(D)J gene recombination (Table 1). The complementarity determining region (CDR) 1 of F8 and 2F5 was identical, but the CDR2 and CDR3 were unique (data not shown).

**Table 1:** Heavy chain and light chain gene families of clone F8 and 2F5 and the V(D)J recombination of each clone.

	VH			VL	
Clone	V-GENE	J-GENE	D-GENE	V-GENE	J-GENE
F8	IGHV3-23*04F	IGHJ4*02 F	IGHD2-2*01 F	IGLV2-14*01 F	IGLJ1*01 F
2F5	IGHV3-30*04 F/IGHV3-30-3*03 F	IGHJ4*02 F	IGHD6-19*01 F	IGLV2-8*01 F	IGLJ2*01 F/IGLJ3*01 F

**Figure 5:**
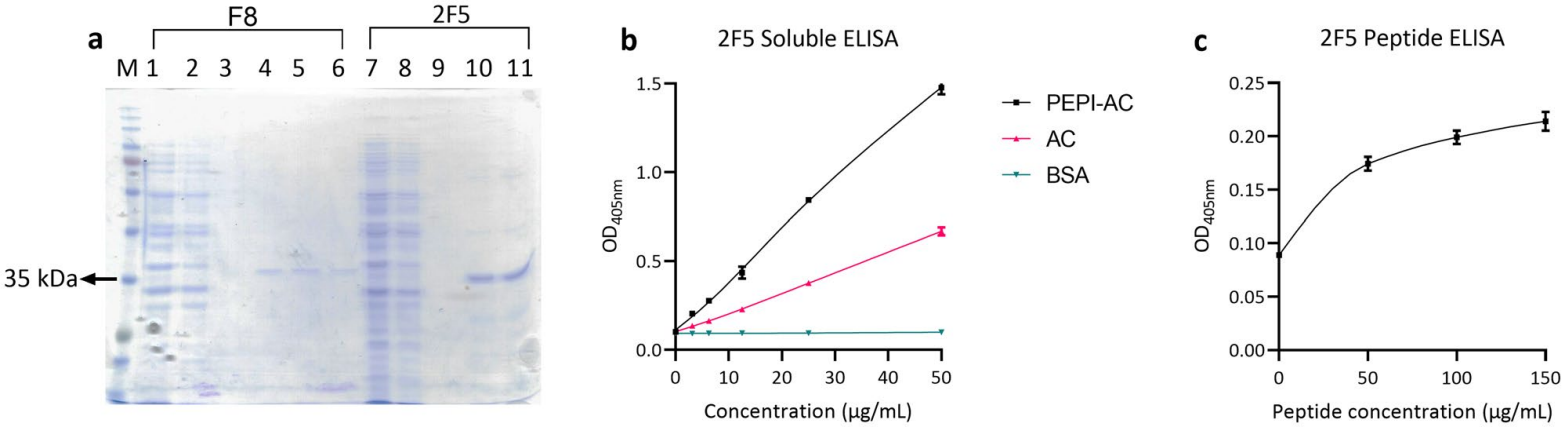
(**a**) Clone F8 and 2F5 with size approximate 35 kDa were purified and analysed on 12% SDS-PAGE. M: Opti-protein XL marker (abm), 1: F8 periplasmic crude, 2: F8 flow-through, 3: F8 wash, 4–6: F8 elution fraction, 7: 2F5 periplasmic crude, 8: 2F5 flow-through, 9: 2F5 wash, 10–11: 2F5 elution fraction. ELISA of the soluble 2F5 against (**b**) PEPI-AC and AC protein, (**c**) PEPITEM peptide. (**b**) In soluble ELISA, PEPI-AC, AC and BSA were coated on wells, 2F5 were added for binding and probed with anti-c-myc HRP. The result showed binding of 2F5 to PEPI-AC at higher signal compared to AC and BSA. (**c**) For peptide ELISA, 2F5 were coated on the wells, PEPITEM peptide were added for binding and probed with streptavidin HRP. The original gel image is presented in Supplementary Fig. S6. All the ELISA data was plotted as mean of triplicate. The error bars indicate the mean ± SD of three independent ELISA reading.

Soluble expressions of clone F8 and 2F5 by HB2151 were purified and analysed on 12% SDS-PAGE (Fig. 5a). Despite the same culture volume, the yield of F8 was significantly lower compared to 2F5 indicating poor solubility. Therefore it was not feasible to analyse clone F8 further. The binding functionality of the soluble 2F5 to PEPI-AC was confirmed through ELISA. A two-fold titration of the 2F5 scFv was conducted and the results showed the binding relation to the scFv concentration. The absorbance signal of 2F5 against PEPI-AC was higher than the readings for AC and BSA (Fig. 5b). The trend of the absorbance readings obtained with the soluble proteins were similar to those obtained by phage ELISA (Fig. 4b). In addition, binding of the clones to the synthesized PEPITEM peptide was tested as well (Fig. 5c). 10 μg of 2F5 was coated and different amounts of peptide were added for binding. Bound peptides were probed with streptavidin-HRP. The results indicates that 2F5 was able to bind to the synthetic PEPITEM peptide well with low non-specific binding to BSA.

## Discussion

Antibody phage display has continuously been used successfully to isolate unique monoclonal antibodies against a wide range of targets. This is mainly due to the robustness of phage particles to withstand harsh conditions to allow many physical alterations to the panning procedures^6,15^. Although antibodies against proteins are routinely reported, the application of antibody phage display for the isolation of antipeptide antibodies are scarce^5^. The success for antipeptide antibody development by animal immunization depends on several factors such as peptide sequence, peptide synthesis, peptide-carrier protein conjugation, the choice of the host animal, and antibody purification^16–18^. The most challenging and important aspect would be the peptide sequence selection. Although several guidelines have being reported to help maximize the development of antipeptide antibodies, the guidelines are still not able to guarantee success. When considering antipeptide antibody development by phage display, the same considerations would take precedence including the length of the peptide^17,18^. The short nature of peptides brings about challenges in terms of presentation and attachment to solid phase for antibody binding during panning campaigns^16^.

In many peptide antibody development projects, synthetic peptides are commonly used via conjugation to a carrier protein or with chemical modifications for tag introduction^17,19–22^. The general concern with such approaches is the potential modification of the peptide structure and its orientation on the carrier protein which could affect its accessibility for binding^23^. In addition, the presence of large carrier proteins could also interfere with the panning process by generating more binders to the carrier protein than the actual peptide itself. Additionally, the factors involved in peptide conjugation would also play a pivotal role as chemical conjugation of the peptide to carrier proteins could result in antigenicity changes to the peptide^9,24^. The conjugation process would also have batch to batch variation in terms of purity and yield leading to inconsistency of the target for selection. Therefore, the possibility of producing the target peptide as a recombinant fusion protein by cloning could help to alleviate this issue. The prokaryotic expression system would allow the generation of the peptide fused to the anchor protein in vivo therefore alleviating concerns of batch-to batch variation and inconsistencies in conjugation ratios.

The use of an immunogenic protein such as AC from Mtb that is of modest size at 16 kDa would make it ideal for expression and presentation. The relatively small size of the anchor protein would make it less likely to compete with the peptide for binding to the antibodies. A flexible glycine-serine linker was used to allow the PEPITEM peptide a greater flexibility for binding to the antibody presenting phage particles. This would overcome the possibility of a rigid folding of the peptide to the anchor protein that could influence the folding and presentation of the peptide. The adaptation of the AC as the anchor protein meant that further modifications was no longer necessary to allow attachment to the solid phase for biopanning. This would also increase the likelihood of the target peptide to be coated to the solid phase. As the production is based on an expression plasmid, swapping of peptides can be done seamlessly to allow presentation of other peptide sequences in the future.

The application of a subtractive panning strategy stems from the need to remove binders against the AC anchor protein. The process was designed with 3 pre-incubations to remove potential binders to the solid phase and blocking agent. These pre-incubations were focused on removing binders to the solid phase, blocking agent, and non-specific interactions. This was followed then by allowing the antibody library pool to interact with the anchor protein (AC) in the well. At this point, the interaction between the antibody presenting phage particles would bind to the AC producing a smaller pool of sieved antibody-phage particles. The low background readings suggest that preincubation of the AC prior to the panning process with PEPI-AC was sufficient to remove any non-specific and AC-specific binders that would otherwise interfere with the panning outcome. It is likely that the use of a larger anchor protein would not be able to produce such an effect as larger protein molecules would likely be over presented to the antibodies making it impossible for the antibody to bind to the peptide sequence.

Removal of the unbound antibody-phage particles to a fresh well with PEPI-AC would then allow for a lesser competition, lower non-specific binding, and allow a concentrated pool of antibody-phage particles to bind to PEPI. This was evident with the enrichment from the panning protocol being visible from round 1 of panning. We hypothesize that the 1 round enrichment could be the consequence of the additional pre-incubation steps and AC binding step that sieved the library diversity leaving behind only a small pool of antibody binders to interact. Therefore, the eluted phage binders would generate a higher degree of enriched PEPITEM antibody phage clones during the rescue. Even if the number of unique phage clones may not be high, the recovery process in the panning protocol would ensure the amplification of these minority clones to generate a strong signal during polyclonal analysis. It is still uncertain if this was the exact cause for a minimal round clonal enrichment. Therefore, additional panning campaigns with other peptide sequence motifs would be carried out in the near future to ascertain if the theory holds merit. Even so, the enrichment pattern from the PEPI-AC panning was sufficient to yield PEPI specific clones as an outcome.

The isolated clone 2F5 was able to bind well to the PEPI-AC construct with low absorbance readings to AC. This indicates that the clones are binding to the PEPI region of the PEPI-AC protein. The signal from Fig. 5c shows lower absorbance reading for AC suggesting that the binding may also include interaction to a portion of AC at the fusion site. A major concern with the proposed approach was the homogeneity of the PEPI peptide presentation as a recombinant fusion towards that of a synthetic peptide. To this end, binding assays were carried out with both the PEPI-AC and the synthesized PEPITEM peptide. The ELISA data shows that both clones were able to bind to the synthetic PEPITEM peptide albeit at a lower absorbance. The lower absorbance reading is expected as binding affinity of the antibody to the synthetic peptide maybe be lower if compared to the fusion protein^3^. We do not discount the possibility that the low absorbance could be due to the low affinity of the antibody clones against the target. As conventional panning strategies normally requires 3 to 5 rounds of enrichment, it is likely that by selecting clones after 1 round of panning may have skewed the selection process to yield clones with lower affinities. Hence, downstream affinity maturation of clones should be investigated to determine the affinity of the clones. However, the ability of 2F5 to bind to the PEPITEM sequence in both peptide and fusion protein configuration suggest the possibility of applying the proposed system to generate antipeptide antibodies.

In conclusion, the application of an anchor protein as a fusion molecule to produce peptide specific targets recombinantly is feasible to be applied for phage display panning. The process also highlights the importance of proper and sufficient pre-incubation to remove non-specific and unrelated binders from the library pool to obtain a satisfactory enrichment during panning. We do not discount the possibility that more panning rounds may be required for other peptide motifs and even to obtain higher affinity antibody clones, but the general concept of the panning process should remain the same. Additionally, the affinity of the isolated clones could be further improved for application in a diagnostic platform. Overall, the proposed panning process can be considered as an alternative approach for anti-peptide antibody development by phage display.

## Materials and methods

### Cloning and expression of AC and PEPI-AC

DNA sequence of PEPITEM corresponding to residue S28-R41 (SVTEQGAELSNEER) was obtained from GenBank (Accession No.: NM_001135699.2) and synthesised as forward and reverse primers. The complementary pairs of primers (PEPITEM_NcoI_Fw: 5′-*C ATG G*CC TCT GTA ACT GAG CAA GGA GCT GAA TTA TCC AAT GAG GAG AGG GT*C*-3′; PEPITEM_XhoI_Rv: 5′-*T CGA G*AC CCT CTC CTC ATT GGA TAA TTC AGC TCC TTG CTC AGT TAC AGA GG*C*-3′) containing NcoI and XhoI restriction site overhang were annealed. Briefly, 2 μg of each primer was mixed in annealing buffer (10 mM Tris, pH8, 50 mM NaCl, 1 mM EDTA) in a total volume of 50 μL. The reaction was incubated in thermocycler programmed to start at 95 °C for 2 min (min) and gradually reduce 10 °C every 5 min to reach final temperature of 25 °C. The annealed primers were diluted with 45 μL of nuclease free water and the concentration was quantified using nanodrop.

The gene of Mtb α-crystalline (AC) was obtained from previous work^25^. The gene of AC was first amplified by PCR and cloned into pLABEL at SalI and NotI restriction site (construct 1). Subsequently, the annealed PEPITEM gene was cloned to 5’ end at NcoI and XhoI restriction site (construct 2). Then, the sequence containing PEPITEM, GS linker and AC gene were cut out with NcoI and NotI and subcloned into a modified pRSET-pelB (construct 3). The design of pRSET-pelB was derived from pRSET-BH6 with the introduction of a pelB leader at the N-terminal of the Avi-tag^25^. This construct would produce PEPI-AC used throughout the experiments. The PEPI-AC construct consist of the pelB, PEPITEM sequence, GS linker, AC, Avi-tag and hexahistidine tag. Concurrently, the GS linker together with AC gene in construct 1 were double digested and subcloned to pRSET-pelB at NcoI and NotI restriction site to yield a similar construct to PEPI-AC excluding the PEPITEM gene (construct 4). The AC construct would then consist of the pelB, immunoglobulin heavy joining 4 (IGHJ4) peptide sequence, GS linker, AC, Avi-tag and hexahistidine tag to function as a background for subtraction. This construct would produce AC used throughout the experiments. All the clones were verified by Sanger sequencing.

pRSET-pelB vector carrying PEPI-AC and AC were transformed into BL21 (DE3) (*fhuA2 [lon] ompT gal (λ DE3) [dcm] ∆hsdS λ DE3* = *λ sBamHIo ∆EcoRI-B int::(lacI::PlacUV5::T7 gene1) i21 ∆nin5*) with pRARE3. The pRARE3 helper plasmid carries the gene for biotin ligase which would aid in the in vivo biotinylation process. The culture was induced with 1 mM IPTG and supplemented with 50 μM of biotin at OD_600nm_ of 0.6. The expression was carried out overnight at 25 °C, 160 rpm. On the next day, the culture was harvested at 8000 × *g*, 10 min and resuspended with lysis buffer (50 mM NaH_2_PO_4_, 300 mM NaCl, 10 mM imidazole, 20 μg/mL lysozyme, pH 7.4). After 1 h (h) incubation on ice, the cell suspension was sonicated (Sonicator 3000, Misonix). The cell lysate was centrifuged at 8000 × *g*, 10 min. The cell pellet was then resuspended with urea buffer (100 mM NaH_2_PO_4_, 10 mM Tris·HCl, 8 M urea, pH 8.0) and incubated at room temperature with rotation for 30 min. Solubilized protein was recovered by centrifuged at 8000 × *g*, 10 min.

Purification was carried out using IMAC technology. Ni–NTA agarose (Qiagen) was set up and equilibrated with urea buffer before the protein was applied to the column. The protein was allowed to bind to the resin at room temperature with rotation for 30 min. The resin was then washed with 10 column volume of wash buffer (100 mM NaH_2_PO_4_, 10 mM Tris·HCl, 8 M urea, pH 6.3) and bound protein was eluted with elution buffer (100 mM NaH_2_PO_4_, 10 mM Tris·HCl, 8 M urea, pH 4.5) at 0.5 mL fraction. The purified fractions were then dialyzed overnight in PBS. The purified protein was analysed on 12% SDS-PAGE stained with Coomassie Blue stain. For western blot, the protein was transferred to nitrocellulose membrane and blocked with PTM (2% (w/v) skim milk in PBST—0.1% (v/v) Tween 20) for 1 h. The membrane was subsequently incubated for 1 h with streptavidin HRP (Invitrogen) at 1:5000 dilution in PTM. The membrane was washed three times with PBST in each interval steps. All the process was carried out at room temperature with gentle shaking. The protein band was visualized by developing the membrane with peroxidase stain DAB kit (Nacalai Tesque). Concentration of the protein was quantified using Bradford assay (Nacalai Tesque).

### Subtractive panning

The naïve phage display library, Human AntibodY LibrarY (HAYLY) ver 2.0 was prepared as described in previous publication^26^. 100 μL of library phage (10^11^ cfu) was pre-incubated with 100 μL of PTM in a well (Corning™ Stripwell™ Microplates) that has been blocked with PTM. Pre-incubation was carried out at room temperature with shaking for 40 min (Well 1). The process was repeated twice (Well 2–3). Subsequently, the library phage was transferred to another well coated with 5 μg of AC protein (Well 4) that has been blocked with PTM prior to the incubation. This subtractive selection process was carried out at room temperature with shaking for 40 min and repeated twice (Well 5–6). Then, the library phage was transferred to the final well coated with 5 μg of PEPI-AC (Well 7) that has been blocked with PTM prior to the incubation. The binding was carried out at room temperature with shaking for 2 h (h). The well was washed 27 times with PBST using plate washer (Wellwash™ Microplate Washer, Thermo Scientific) and eluted with 100 μL of trypsin (10 μg/mL). Elution was carried out at 37 °C for 30 min. 200 μL of TG1 (*supE thi-1 Δ(lac-proAB) Δ(mcrB-hsdSM)5(rK* − *mK *−*)* [F' *traD36 proAB lacI*^*q*^*Z ΔM15]*) at OD_600nm_ of 0.5 was then added to the eluted phage for infection to carry out at 37 °C for 30 min. 10 μL of infected culture was used for titer and the remaining culture was plated out on 2 × YT-amp agar plate (2 × YT agar plate supplemented with 0.1 mg/mL of ampicillin and 2% (w/v) glucose) and incubated overnight at 37 °C.

On the next day, colonies on agar plate were scraped with 2 × YT-amp (2 × YT supplemented with 0.1 mg/mL ampicillin and 2% (w/v) glucose). A 10 mL culture with starting OD_600nm_ of 0.2 was set up and cultured for 1 h at 37 °C, 200 rpm to reach OD_600nm_ of 0.5 prior to infection with M13KO7 helper phage (10^11^ cfu). After 30 min of infection at 37 °C, the culture was centrifuged down at 3500 × *g*, 10 min, and resuspended with 8 mL of 2 × YT-amp/kan (2 × YT supplemented with 0.1 mg/mL ampicillin and 0.06 mg/mL kanamycin) and cultured overnight at 30 °C, 180 rpm. On the next day, the culture was centrifuged at 10,000 × *g* for 10 min and the supernatant was incubated with 2 mL of PEG/NaCl solution (20% (w/v) PEG 6000, 2.5 M NaCl) on ice for 1 h. The mixture was centrifuged at 10,000 × g for 30 min at 12 °C and the pellet was resuspended with 300 μL of PBS. 10 μL of the phage was used for titer and 100 μL of the phage was used for polyclonal ELISA.

Polyclonal ELISA was performed as described in previous publication^26^. Briefly, 50 μL of phage was mixed with 50 μL of PTM and incubated in well coated with 5 μg of AC, PEPI-AC and BSA, respectively. The wells were blocked with PTM and washed three times with PBST prior to phage binding. After 2 h of binding, the wells were washed with PBST for 10 times and subsequently incubated with anti-M13 HRP (GE Healthcare, 1:5000 dilution in PTM) for 1 h. The wells were developed with 2,2′-azino-bis(3-ethylbenzothiazoline-6-sulphonic acid) (ABTS) in dark after three PBST wash. Absorbance reading at 405 nm was recorded using spectrophotometer (MultiSkan™ GO, Thermo Scientific). All the steps were performed at room temperature with gentle shaking.

### Selection of monoclonal antibodies against PEPI-AC

10^8^ cfu of phage was pre-incubated in a well coated with 5 μg of AC. The well was blocked with PTM prior to the 1 h pre-incubation at room temperature with shaking. The phage was then serial diluted and used to infect TG1 and XL1-Blue MRF’ (*recA1 endA1 gyrA96 thi-1 hsdR17 supE44 relA1 lac* [F´ *proAB lacI*^*q*^* Z*∆*M15* Tn*10* (Tet^r^)]). 92 colonies were selected respectively and subjected to monoclonal phage ELISA as described previously^26^. Briefly, the colonies were cultured, and monoclonal phages were packaged in microtiter culture plate. To perform ELISA, 1 μg of PEPI-AC, AC and BSA were coated, respectively, on 96-well microtiter plates. The antigen-coated microtiter plates were blocked with PTM for 1 h before the monoclonal phages were transferred to the wells for binding. Subsequent to the 2 h binding, the wells were washed with PBST and anti-M13 HRP (1:5000 dilution in PTM) was added to incubate for 1 h. Each interval steps were washed three times with PBST using plate washer. All the steps were carried out at room temperature with shaking. The signal was developed using ABTS in dark. The absorbance reading at 405 nm was recorded using spectrophotometer.

Positive clones were subjected to colony PCR and clones with correct band size (1200 base pair) were cultured overnight. The phagemids were extracted and sent for sequencing. The DNA sequences were analysed on IMGT/V-quest (www.imgt.org/IMGT_vquest/vquest).

### Expression of soluble monoclonal antibody

The clone F8 and 2F5 were transformed to expression strain, HB2151 (K12, *ara ∆(lac-proAB) thi/F’ proA* + *B lacI*^*q*^* lacZ∆M15*). The antibody clones were expressed with a hexahistidine and c-myc tag at the C-terminal end. The culture was induced with 1 mM IPTG at OD_600nm_ of 0.6 and expression was carried out overnight at 25 °C, 160 rpm. On the next day, the culture was harvested at 8000 × *g*, 10 min (min). The pellet was resuspended with ice-cold TES buffer (0.2 M Tris·HCl, 0.5 mM EDTA, 0.5 M sucrose, pH 8.0) and followed by addition of ice-cold 1:5 TES (TES buffer diluted at 1:5 ratio with distilled water). After 1 h (h) incubation on ice, the cell suspension was centrifuged at 8000 × *g*, 10 min. The supernatant was recovered and subjected to IMAC purification. Ni–NTA agarose (Qiagen) were set up and equilibrated with binding buffer (20 mM NaH_2_PO_4_, 500 mM NaCl, 20 mM imidazole, pH 7.4) before the protein was applied to the column. The protein was allowed to bind to the resin at room temperature with rotation for 1 h. The resin was then washed with 10 column volume of binding buffer and bound protein was eluted with elution buffer (20 mM NaH_2_PO_4_, 500 mM NaCl, 500 mM imidazole, pH 7.4) at 1 mL fraction. The purified protein was analysed on 12% SDS-PAGE stained with Coomassie Blue stain. Concentration of the protein was quantified using Bradford assay (Nacalai Tesque).

### Soluble ELISA

1 μg of PEPI-AC, AC and BSA were coated (100 μL) overnight. The wells were blocked with PTM for 1 h. Soluble scFv was serial diluted and added to each well and incubated for 2 h. The binding of the scFv was probed using anti-c-myc HRP (1:5000 dilution in PTM) for 1 h and developed with ABTS. The wells were washed three times with PBST in between all the steps. All the processes were performed at room temperature with gentle shaking.

### Peptide ELISA

PEPITEM peptide was synthesised with biotin and alanine linker at the N-terminal (N-terminal—Biotin—AAAAAASVTEQGAELSNEER—NH2—C-terminal). 10 μg of scFv was coated (100 μL) overnight. The wells were blocked with PTM for 1 h. 5–15 μg of synthesized PEPITEM peptide (dissolved in PBS) was added to respective well and incubated for 2 h. PBS was added in replace of PEPITEM peptide in control well. The binding of the peptide to the coated scFv was probed using streptavidin HRP (1:5000 dilution in PTM) for 1 h and developed with ABTS. The wells were washed three times with PBST in between all the steps. All the processes were performed at room temperature with gentle shaking.

### Supplementary Information

Supplementary Information.

## Data Availability

The datasets generated and analysed during the current study are available from the corresponding author on reasonable request.
