# Supplementary material for: Subtractive panning for the isolation of monoclonal PEPITEM peptide antibody by phage display
Source: Sci Rep. 2023 Aug 21;13:13627. doi: 10.1038/s41598-023-40630-7 (PMC10442400; doi:10.1038/s41598-023-40630-7)
Supplement: Supplementary file 1 — Supplementary Information. [file 41598_2023_40630_MOESM1_ESM.docx]

**SUPPLEMENTARY INFORMATION**

**Subtractive Panning for the Isolation of Monoclonal PEPITEM Peptide Antibody by Phage Display**

**Mohammed Al-Assiri ^1,2,*^, Jing Yi Lai ^3^, Angela Chiew Wen Ch’ng ^3^, Yee Siew Choong ^3^, Asma Alanazi ^2,4^ and Theam Soon Lim ^3,5,*^**

^1^Department of Basic Sciences, College of Science and Health Professions, King Saud bin Abdulaziz University for Health Sciences (KSAU-HS), Riyadh, KSA

^2^King Abdullah International Medical Research Center (KAIMRC), Riyadh, KSA

^3^Institute for Research in Molecular Medicine, Universiti Sains Malaysia, 11800 Penang, Malaysia

^4^Department of Basic Medical Sciences, College of Medicine, King Saud bin Abdulaziz University for Health Sciences (KSAU-HS), Riyadh, KSA

^5^Analytical Biochemistry Research Centre, Universiti Sains Malaysia, 11800 Penang, Malaysia

*Correspondence: assirim@ksau-hs.edu.sa (M.A.); [theamsoon@usm.my](mailto:theamsoon@usm.my) (T.S.L.)

**Supplementary Figures**

**
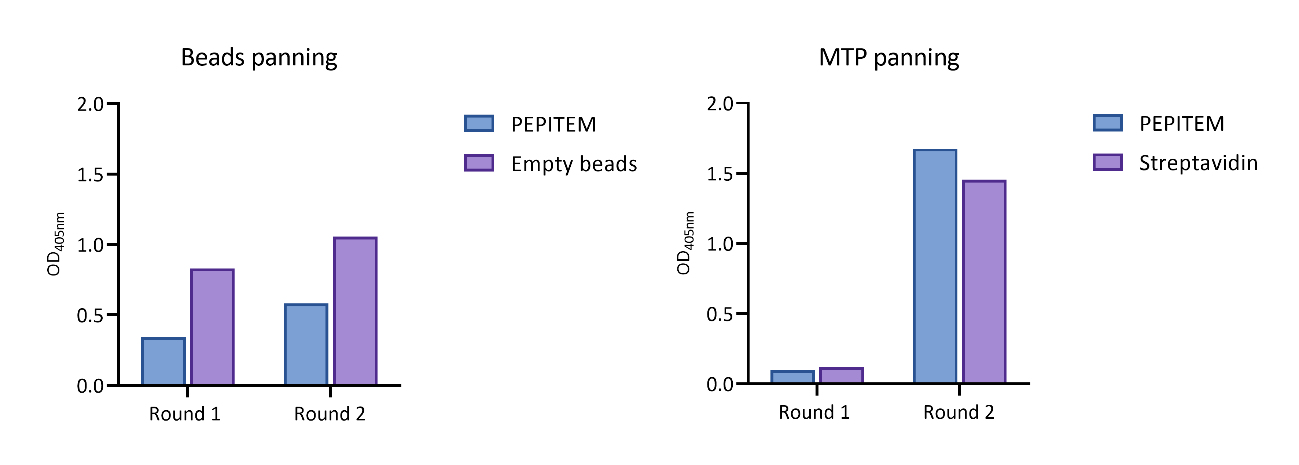
**

**Figure S1.** Panning approaches using streptavidin magnetic beads and streptavidin coated microtiter plate (MTP) well were carried out. The results showed unsuccessful enrichment of the PEPITEM binders. In beads panning, the signal of background was high, which is 0.831 and 1.056 for round 1 and round 2 respectively compared to PEPITEM conjugated beads which give a reading of 0.345 and 0.583 for round 1 and round 2 respectively. For MTP panning, polyclonal phage ELISA showed signal of 0.099 and 1.675 for round 1 and round 2 respectively, however background binder to the streptavidin coated well was enriched, with the signal increased from 0.120 to 1.455 from round 1 to round 2.


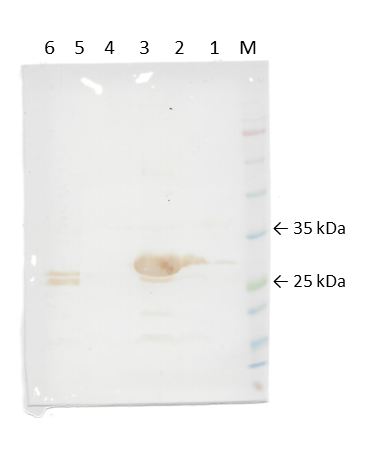


**Figure S2.** Expression of PEPI-AC and AC. (a) Western blot of the protein extracted from different fraction. M: Opti-XL protein marker (abm), 1: PEPI-AC periplasmic crude, 2: PEPI-AC cytoplasmic crude, 3: PEPI-AC pellet, 4: AC periplasmic crude, 5: AC cytoplasmic crude, 6: AC pellet. The proteins were probed with Streptavidin HRP (1:5000).


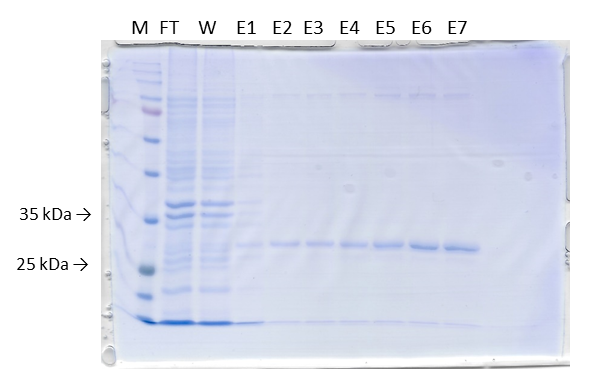


**Figure S3.** Purification of PEPI-AC analysed on 12% SDS-PAGE. M: Opti-protein XL marker (abm), FT: flow-through, W: wash, E1-7: elution fraction 1-7.


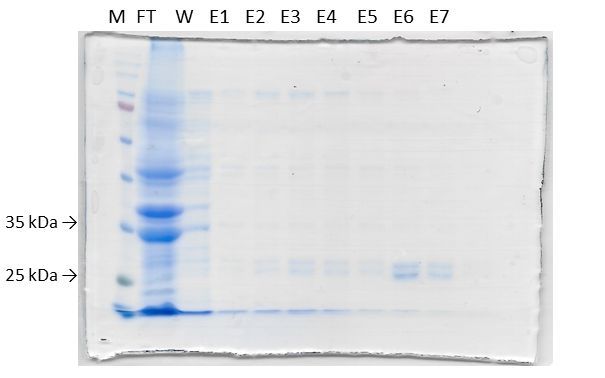


**Figure S4.** Purification of AC analysed on 12% SDS-PAGE. M: Opti-protein XL marker (abm), FT: flow-through, W: wash, E1-7: elution fraction 1-7.


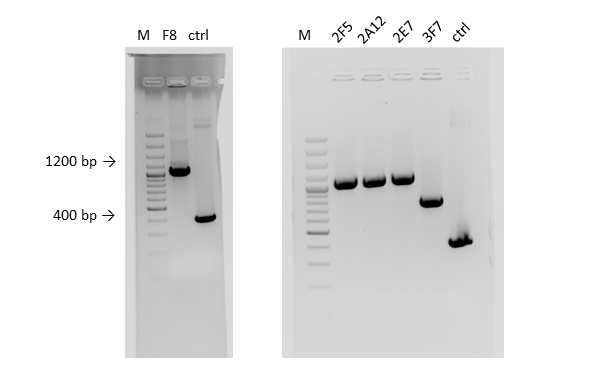


**Figure S5.** The clones were subjected to colony PCR and showed that all clones containing full scFv size (1200 bp), except for clone 3F7 which is only half of the size. M represents 100 bp plus DNA ladder (Thermo Scientific) and ctrl represents the pLABEL vector.


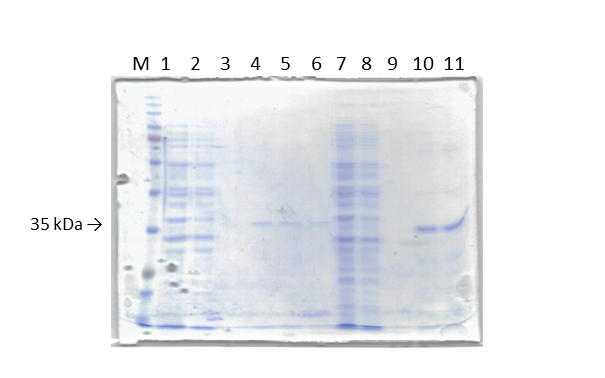


**Figure S6.** Clone F8 and 2F5 with size approximate 35 kDa were purified and analysed on 12% SDS-PAGE. M: Opti-protein XL marker (abm), 1: F8 periplasmic crude, 2: F8 flow-through, 3: F8 wash, 4-6: F8 elution fraction, 7: 2F5 periplasmic crude, 8: 2F5 flow-through, 9: 2F5 wash, 10-11: 2F5 elution fraction.

**Supplementary Methods**

**Beads panning**

The synthesized PEPITEM peptide diluted in PBS (10 μg) was conjugated overnight at 4ºC with rotation to 100 μL of streptavidin magnetic beads (Dynabeads, Invitrogen). The bead was washed on the next day with PBST and stored in 100 μL of PBS. The panning and polyclonal ELISA was carried out using semi-automated approach as described by Ch'ng, et al. ^1^. Prior to panning, 100 μL of library phage (10^11^ cfu) was pre-incubated with 100 μL of PTM and 10 μL of blank streptavidin beads in an immunotube (SPL Life Sciences) that has been blocked with PTM. Pre-incubation was carried out at room temperature with rotation for 1 h. The blank streptavidin beads were then removed, and the pre-incubated phage was mixed with 20 μL of PEPITEM-conjugated beads for panning process using the semi-automated protocol. Two rounds of panning were conducted and the amplified phage from each panning round was subjected to polyclonal phage ELISA to check enrichment of the binders.

**Microtiter plate (MTP) panning**

MTP panning was carried out as described previously with some modification ^2^. Briefly, 200 ng of streptavidin (Sigma) was coated on microtiter plate well overnight at 4ºC. On the next day, the wells were washed 3 times with PBST (0.1%(v/v) Tween 20), followed by blocking with 2% BSA-PBST for 1 h. After 3 PBST wash, 1 μg of peptide was loaded to the well. Concurrently, 100 μL of library phage (10^11^ cfu) was pre-incubated with 100 μL of 2% BSA-PBST in a blocked streptavidin well. After 1 h of pre-incubation, the library phage was transferred to the well coated with PEPITEM peptide. The phage was allowed to bind for 2 h before washing 10 times with PBST using plate washer (Wellwash^TM^ Microplate Washer, Thermo Scientific). All the steps were carried out at room temperature with shaking. Phage eluted by 100 μL of trypsin was used to infect 200 μL of TG1 at OD_600nm_ of 0.5 for 30 min at 37ºC. 10 μL of infected culture was used for titre while remaining of the culture was supplemented with 2% (w/v) glucose and 100 μg/mL ampicillin to continue culture for another 2 h. The culture was then co-infected with 10^9^ cfu M13KO7 helper phage at 37ºC for 30 min. The culture was pelleted down and resuspended with 200 μL of 2× YT supplemented with 100 μg/mL ampicillin and 60 μg/mL kanamycin. The phage was allowed to package overnight at 30ºC. On the next day, the culture was pelleted down and 100 μL of the phage was used for second round of panning.

For polyclonal ELISA, 50 μL of phage was mixed with 50 μL of 2% BSA-PBST and incubated in well coated with 200 ng streptavidin and 1 μg of PEPITEM peptides and BSA, respectively. The wells were blocked with 2% BSA-PBST and washed three times with PBST prior to phage binding. After 2 h of binding, the wells were washed with PBST for 10 times and subsequently incubated with anti-M13 HRP (GE Healthcare, 1:5000 dilution in 2% BSA-PBST) for 1 h. The wells were developed with 2,2'-azino-bis(3-ethylbenzothiazoline-6-sulphonic acid) (ABTS) in dark after three PBST wash. Absorbance reading at 405 nm was recorded using spectrophotometer (MultiSkan^TM^ GO, Thermo Scientific). All the steps were performed at room temperature with gentle shaking.

**References**

1 Ch'ng, A. C. W., Konthur, Z. & Lim, T. S. in *Methods in Enzymology* Vol. 630 (ed Challa V. Kumar) 159-178 (Academic Press, 2020).

2 Mohd Ali, M. R. *et al.* Development of monoclonal antibodies against recombinant LipL21 protein of pathogenic Leptospira through phage display technology. *International Journal of Biological Macromolecules* **168**, 289-300 (2021). <https://doi.org:https://doi.org/10.1016/j.ijbiomac.2020.12.062>
